# AMPK maintains energy homeostasis and survival in cancer cells via regulating p38/PGC-1*α*-mediated mitochondrial biogenesis

**DOI:** 10.1038/cddiscovery.2015.63

**Published:** 2015-12-21

**Authors:** B Chaube, P Malvi, S V Singh, N Mohammad, B Viollet, M K Bhat

**Affiliations:** 1 National Centre for Cell Science, Savitribai Phule Pune University Campus, Ganeshkhind, Pune, Maharashtra 411 007, India; 2 INSERM, U1016, Institut Cochin, Paris, France; 3 CNRS, UMR 8104, Paris, France; 4 Université Paris Descartes, Sorbonne Paris Cité, Paris, France

## Abstract

Cancer cells exhibit unique metabolic response and adaptation to the fluctuating microenvironment, yet molecular and biochemical events imprinting this phenomenon are unclear. Here, we show that metabolic homeostasis and adaptation to metabolic stress in cancer cells are primarily achieved by an integrated response exerted by the activation of AMPK. We provide evidence that AMPK-p38-PGC-1*α* axis, by regulating energy homeostasis, maintains survival in cancer cells under glucose-limiting conditions. Functioning as a molecular switch, AMPK promotes glycolysis by activating PFK2, and facilitates mitochondrial metabolism of non-glucose carbon sources thereby maintaining cellular ATP level. Interestingly, we noted that AMPK can promote oxidative metabolism via increasing mitochondrial biogenesis and OXPHOS capacity via regulating expression of PGC-1*α* through p38MAPK activation. Taken together, our study signifies the fundamental role of AMPK in controlling cellular bioenergetics and mitochondrial biogenesis in cancer cells.

## Introduction

Altered glucose metabolism is a characteristic feature of rapidly growing cancer cells.^1,2^ Cancer cells metabolize glucose primarily through aerobic glycolysis.^2^ Enhanced glycolysis is considered as one of the hallmarks of aggressive tumors. Nonetheless, recently, it has been shown that functional mitochondria are vital for tumorigenesis.^3–6^ Unlike normal tissues, tumors have more dense structure and irregular distribution of blood vessels owing to the immense ability of cancer cells to proliferate. In solid tumors, various stress conditions like low nutrient availability, energy depletion, hypoxia and oxidative stress arise during excessive growth and proliferation.^7^ Owing to the heterogeneous distribution of oxygen, glucose, glutamine and other nutrients in the solid tumor, cells have to adapt to nutritionally stressed microenvironment which confers selective survival advantage. A question still remains to be answered as to how cancer cells cope up with these tribulations to achieve survival, and simultaneously maintain rapid growth and proliferation. Given the heterogeneous nature of tumor microenvironment, there must be adaptive mechanisms that can maintain energy and metabolic homeostasis. Unfortunately, the nature of actual metabolic remodeling in cancer cells has often been veiled owing to the use of *in vitro* cell culture condition that provides high glucose and oxygen in contrary to the actual situation found in tumor microenvironment. It is well known that chronic energy deprivation and metabolic stress results in elevated mitochondrial oxidative capacity in muscles cells by inducing mitochondrial biogenesis.^8–10^ However, in cancer cells, despite high levels of physiological stress, the role of mitochondria in maintaining cell survival and homeostasis is not very clear.

All the cells have specific energy and nutrient sensors like AMP-activated protein kinase (AMPK) and mammalian target of rapamycin (mTOR). AMPK, upon energy depletion, initiates signaling cascade resulting in the suppression of ATP consuming pathways with concomitant induction of biochemical reactions that generate ATP.^11^ AMPK serves as a fuel gauge as it is activated by low ATP/AMP ratio, and is thought to protect mammalian cells against energy deprivation by controlling various pathways to maintain energy homeostasis.^12^ Conversely, mTOR is a master regulator of cell growth and proliferation under nutrient-abundant conditions.^13^ AMPK is known to inhibit mTOR by directly phosphorylating raptor, one of the molecules of TOR complex.^11,13^ Most of the reports suggest that AMPK is a tumor suppressor as it inhibits many pathways involved in growth and proliferation.^11^ Other than regulating metabolism, it is also believed to regulate expression of genes associated with metabolism via localizing to the nuclei of many cells.^14,15^ Recent correlative studies suggest that AMPK increases mitochondrial biogenesis^8^ and OXPHOS capacity^16^ in rat skeletal muscles. It has been shown that peroxisome proliferator-activated receptor coactivator-1 (PGC-1*α*) is involved in mitochondrial biogenesis in muscles.^17,18^ However, the mechanisms controlling mitochondrial biogenesis and cell survival under metabolic stress in cancer cells are not very clear. To examine whether AMPK is involved in mitochondrial biogenesis so as to provide survival advantage to cancer cells under glucose-limiting conditions, we used both biochemical as well as genetic approaches.

Herein, we describe that activation of AMPK is required for increased mitochondrial biogenesis in response to glucose limitation. Furthermore, we show that AMPK activation is required for increased expression of PGC-1*α* and TFAM. Expression of PGC-1*α* is controlled by AMPK-induced activation of p38MAPK. Overall, this study highlights the role of AMPK in controlling cellular bioenergetics and mitochondrial biogenesis in cancer cells under glucose-limiting conditions.

## Results

### AMPK protects cancer cells from glucose deprivation-induced death

**Figure 1:**
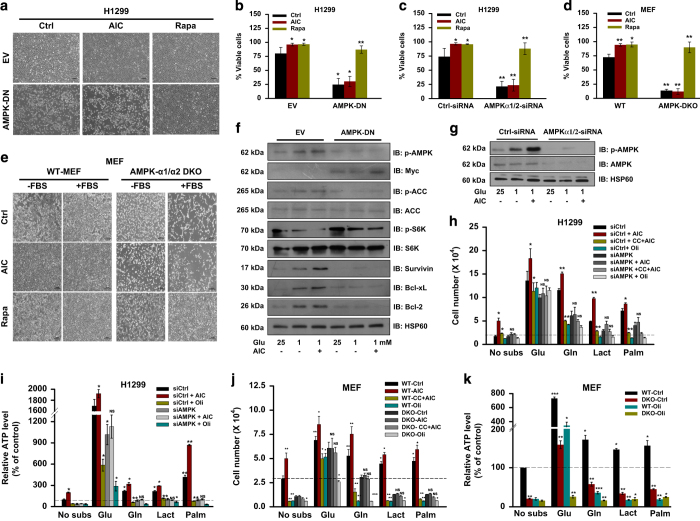
AMPK maintains cell survival under metabolic stress by promoting metabolism of non-glucose carbon sources. (**a**) Images representing the cellular morphology of H1299 cells stably transfected with either pCDNA3 vector (H1299-EV) or with dominant-negative form of AMPK-*α*1 subunits tagged with Myc (H1299-DN). These cells were grown under glucose-limiting conditions (1 mM glucose, 2 mM glutamine and 10% FBS) in the presence or absence of 0.5 mM AICAR (AIC) or 20 nM rapamycin (Rapa) for 36 h. (Scale bar, 50 *μ*m). (**b**) Viability of H1299-EV and H1299-DN cells cultured under the conditions mentioned in **a** for 36 h, as assessed by PI staining. (**c**) H1299 cells were transfected with AMPK-*α*1/2-specific siRNA and grown under conditions used in **b**, viability of these cells was assessed by PI staining. (**d**) Viability of AMPK-*α*1/2^+/+^ (WT) and AMPK-*α*1/2^−/−^ (DKO) MEFs cultured under the conditions mentioned in **a** for 36 h, as assessed by PI staining. (**e**) Representative images showing cellular morphology of WT and AMPK-DKO MEFs cultured in DMEM with or without glucose and FBS in the presence or absence of 0.5 mM AICAR or 20 nM rapamycin for 36 h (Scale bar, 50 *μ*m). (**f**) H1299-EV and H1299-DN cells were grown in either 25 or 1 mM glucose in the presence or absence of 0.5 mM AICAR for 24 h. Representative immunoblots showing the levels of indicated molecules. Antibody for Myc tag (9E10) was used for detecting overexpressed dominant-negative form of AMPK *α*1 subunit. HSP60 was used as a loading control. (**g**) Representative immunoblots showing phosphorylated and basal levels of AMPK in H1299 cells transfected with either control or AMPK-*α*1/2-specific siRNAs. HSP60 was used as loading control. (**h**) Survival and (**i**) ATP level in H1299 cells (transfected with AMPK-*α*1/2 siRNA) cultured in glucose and glutamine-free DMEM supplemented with 5 mM glucose (Glu), 5 mM glutamine (Gln), 5 mM galactose (Gal), 10 mM lactate (Lact) and 100 *μ*M palmitate (Palm) in the presence or absence of 0.5 mM AICAR (AIC), 10 *μ*M compound C (CC) and 1 *μ*M oligomycin (Oli) for 48 h. (**j**, **k**) Survival and relative ATP level in WT and DKO MEFs cultured under similar conditions used in **h** and **i**. Values are represented as mean±S.D. *P<0.05, **P<0.01, ***P<0.001 denote significant differences between the groups.

Considering the heterogeneity and physiological stress in tumor microenvironment, we hypothesized that under metabolic stress, cells survive by activating AMPK to maintain energy and metabolic homeostasis. To investigate the involvement of AMPK in cell survival, we used H1299 cells stably transfected with dominant negative form of AMPK-*α*1 subunit (H1299-DN). We observed that H1299-DN cells failed to survive under glucose-limiting conditions despite the activation of AMPK by AICAR as compared with H1299-EV cells (Figures 1a and b). This finding was consistent with siRNA-mediated knockdown of AMPK-*α*1/2 in H1299 cells (Figure 1c). Further, to confirm the cell-protecting role of AMPK, we used transformed AMPK-*α*1/*α*2 double knockout (AMPK-DKO) MEFs. In parallel, matching wild-type MEF (WT-MEF) were used as control. WT-MEF treated with AICAR survived under nutritional stress, whereas DKO cells failed to survive even upon AICAR treatment (Figures 1d and e, and Supplementary Figures S1a–e). As AMPK is known to suppress mTOR that is involved in cell proliferation, we hypothesized that inhibition of mTOR might support cell survival under glucose-limiting conditions. Irrespective of AMPK status, rapamycin (an inhibitor of mTOR) promoted survival in H1299 as well as MEF cells under metabolic stress (Figures 1a–e).

Further, we checked molecular events involved in the survival of H1299-EV or H1299-DN cells grown in 1 mM glucose in the presence or absence of AICAR. We found that glucose-limiting condition activated AMPK, which was further augmented upon treatment with AICAR in H1299-EV cells in comparison with H1299-DN cells as reflected by phosphorylated levels of ACC (Figure 1f). Using AMPK-*α*1/2-specific siRNA, we observed similar trend in the activation of AMPK by AICAR and/or glucose-limiting conditions (Figure 1g). Moreover, the activation of AMPK resulted in suppression of mTOR pathway as evident by decreased phosphorylation of p70-S6Kinase (Figure 1f). Next, we checked the levels of molecules associated with the survival of H1299-EV and H1299-DN cells grown under glucose-limiting conditions. Increase in protein levels of survivin, Bcl-xL and Bcl-2 was detected in H1299-EV cells under glucose-limiting conditions, which was further elevated upon AICAR treatment, whereas no change in these molecules was observed in H1299-DN cells under similar conditions (Figure 1f). These results indicate that AMPK-mediated cell survival is dependent on inhibition of mTOR.

### AMPK facilitates mitochondrial metabolism of non-glucose substrates under metabolic stress

Balance between energy generation and utilization dictates cell survival under limiting nutrient availability or metabolic stress.^19^ When glucose availability is limited or diminished, cancer cell utilizes non-glucose carbon sources like glutamine, lactate and fatty acids. We asked whether AMPK is involved in facilitating metabolism of these substrates under glucose-deprived conditions. Noticeably, AMPK knocked-down H1299 cells failed to grow in the presence of glutamine, lactate and palmitate in the absence of glucose as compared with control cells (Figure 1h). Treatment with AICAR increased survival, which was reversed by the addition of compound C in control H1299 cells as compared with siAMPK cells (Figure 1h). With the presumption that maintenance of energy balance is the first step associated with AMPK-mediated cell survival, we measured ATP level under varying nutritional status. We noticed increased ATP level in cancer cells upon AMPK activation. Under identical conditions, H1299 (siCtrl) cells treated with AICAR generated more ATP in the presence of glutamine, lactate and palmitate, which is reflected by prolonged survival as compared with cells upon AMPK knockdown (Figures 1h and i; Supplementary Figures S2a and b, S3a–c and S4). Similarly, increase in survival and ATP level was detected in WT-MEFs grown in the presence of these substrates together with AICAR as compared with DKO MEFs (Figures 1g–i). Pharmacological inhibition of enzymes involved in the metabolism of lactate and glutamine by oxamate and 6-diazo-5-oxo-L-norleucine (DON) or epigallocatechin 3-gallate (EGCG), respectively, resulted in reduced cell survival even upon activation of AMPK (Supplementary Figures S2a–d).

Next to determine as to how AMPK facilitates cell survival in the presence of these substrates, we measured ATP level in the presence or absence of oligomycin, an ATP synthase inhibitor. Oligomycin reduced cell survival in the presence of non-glucose substrates, whereas under normal (glucose abundance) conditions oligomycin did not affect cell survival (Figures 1h and j; Supplementary Figure S4), which is correlated with their respective ATP levels (Figures 1i and k). These results suggest that AMPK is involved in promoting metabolism of non-glucose substrate to maintain energy homeostasis and cell survival.

### AMPK induces mitochondrial biogenesis and OXPHOS activity under glucose-limiting conditions

**Figure 2:**
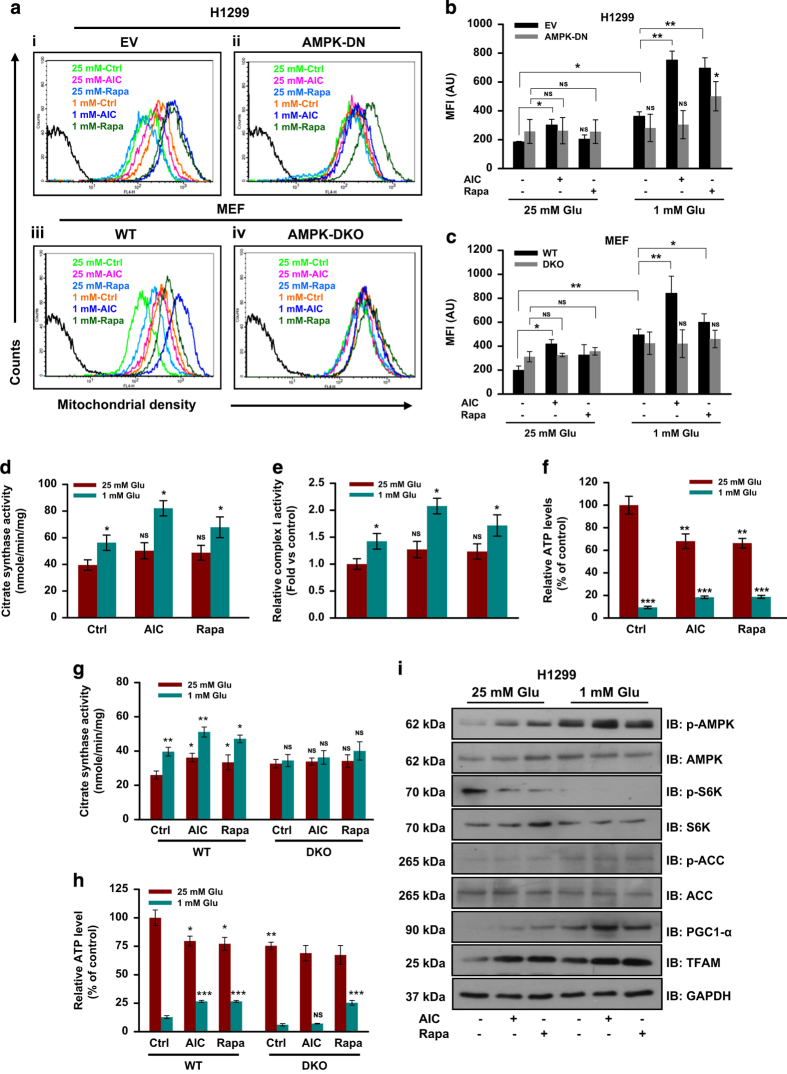
AMPK increases mitochondrial density in cancer cells. (**a**) H1299-EV (i), H1299-DN (ii), WT (iii) and AMPK-DKO (iv) cells were cultured in either 25 mM or 1 mM glucose, and 2 mM glutamine in the presence of 0.5 mM AICAR or 20 nM rapamycin for 24 h. Mitochondrial density was measured using MitoTracker Red FM by flow cytometry. (**b**, **c**) Quantitative bar graphs showing the relative change in the mean fluorescence intensity of MitoTracker Red FM in H1299 cells (**b**) and MEFs (**c**) under indicated treatment conditions. (**d**–**f**) Citrate synthase activity (**d**), respiratory complex I activity (**e**) and relative ATP level (**f**), in H1299 cells grown in DMEM containing 25 or 1 mM glucose with or without AICAR and rapamycin for 24 h. (**g**, **h**) Citrate synthase activity (**g**) and relative ATP level (**h**), in WT and AMPK-DKO MEFs cultured under indicated conditions. (**i**) Immunoblots showing protein levels of indicated molecules involved in AMPK/mTOR pathway and mitochondrial biogenesis. Values are represented as mean±S.D. *P<0.05, **P<0.01 and ***P<0.001 denote significant differences between the groups.

For cancer cells to utilize non-glucose carbon sources such as lactate and glutamine, functional mitochondria are required. As AMPK regulates mitochondrial biogenesis in skeletal muscle upon chronic exercise or energy deprivation,^16,20–22^ we sought to explore whether AMPK is also involved in mitochondrial biogenesis in cancer cells. To confirm this, biochemical and genetic tools were utilized. We noticed that H1299-EV and WT-MEFs grown under glucose-limiting conditions showed high mitochondrial density as compared with those cultured in glucose-abundant conditions (Figures 2a–c). Both AICAR and rapamycin increased mitochondrial density in H1299-EV cells and WT-MEFs under glucose-limiting conditions (Figures 2a–c). However, we did not observe significant change in mitochondrial density in H1299-DN and AMPK-DKO grown under these conditions (Figures 2a–c). Also, increased mitochondrial density was detected in WT-MEF and H1299-EV cells treated with AICAR together with rapamycin as compared with single agent alone (Supplementary Figures S5a and b), whereas no such change was observed in DKO and H1299-DN cells grown under similar conditions (Supplementary Figures S5a and b).

Further, to confirm these observations, we checked citrate synthase and respiratory complex I activity. We found increased citrate synthase and complex I activity in H1299 cells under glucose-limiting conditions, which was further increased upon AICAR or rapamycin treatment (Figures 2d and e). Under glucose-limiting conditions, decrease in ATP levels was detected that increased upon AICAR and rapamycin treatments (Figure 2f). Similar observations were also noted in WT-MEFs. However, in AMPK-DKO cells, irrespective of glucose concentrations as well as AICAR or rapamycin treatment, citrate synthase activity remained unchanged (Figure 2g). In WT-MEFs, relative ATP level was increased upon AICAR and rapamycin treatment under glucose-limiting conditions. However, in AMPK-DKO cells, only rapamycin increased the ATP level under similar conditions (Figure 2h).

Next, we checked molecular events involved in the mitochondrial biogenesis. Increased activation of AMPK and consequent alteration in phosphorylated ACC and p70-S6Kinase were detected in H1299 cells cultured in glucose-limiting conditions (Figure 2i). Under these conditions, pronounced elevation in the levels of proteins regulating mitochondrial biogenesis (PGC-1*α* and TFAM) was observed. AICAR treatment further increased the activation of AMPK and levels of PGC-1*α* and TFAM (Figure 2i). Interestingly, we also observed increased level of PGC-1*α* and TFAM in H1299 cells upon rapamycin treatment under both glucose-abundant and -limiting conditions (Figure 2i). These results indicate that AMPK maintains energy homeostasis under glucose-limiting conditions by promoting mitochondrial biogenesis.

### AMPK-induced mitochondrial biogenesis is mediated by p38-dependent regulation of PGC-1*α*

**Figure 3:**
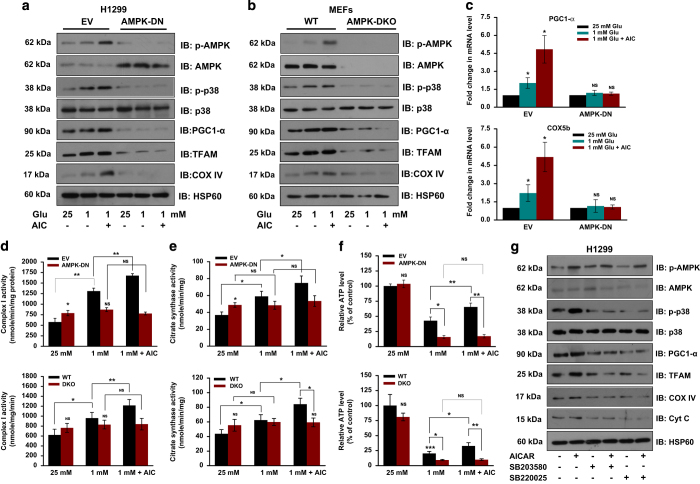
AMPK regulates p38-dependent expression of PGC-1*α*. (**a**, **b**) Immunoblots showing levels of indicated molecules in H1299-EV and H1299-DN cells (**a**) and in MEFs (**b**) cultured in 25 or 1 mM glucose with or without 0.5 mM AICAR for 24 h. HSP60 was used as loading control. (**c**) Relative expression of PGC-1*α* and COX5b mRNA in H1299-EV and H1299-DN cells grown under the conditions used in **a** as evaluated by qRT-PCR. (**d**–**f**) H1299-EV, H1299-DN, WT-MEF and DKO cells were grown under conditions mentioned in **a**. Respiratory complex I activity (**d**), citrate synthase activity (**e**) and relative levels of ATP (**f**) were measured as described in Materials and Methods. (**g**) Immunoblots of indicated molecules in H1299 cells cultured in glucose-limiting conditions with or without 0.5 mM AICAR and 10 *μ*M p38-specific inhibitors, SB203508 and SB220025 for 24 h. Values are represented as mean±S.D. *P<0.05, **P<0.01 and ***P<0.001 denote significant differences between the groups.

As PGC-1*α* and TFAM are involved in mitochondrial biogenesis, we, therefore, explored the upstream events regulating these proteins. It is reported that p38 activates PGC-1*α*, and AMPK is known to activate p38 under chronic energy deprivation.^23–27^ Thus, to investigate whether AMPK can activate p38 in cancer cells, we checked levels of phosphorylated p38 (p-p38) in H1299-EV, H1299-DN, WT-MEFs and AMPK-DKO cells grown under glucose-limiting conditions. Increased levels of p-p38 was detected in H1299-EV as well as in WT-MEFs exposed to low glucose or treated with AICAR. An increase in the levels of mitochondrial markers (PGC-1*α*, TFAM and COX IV) was also observed under similar conditions (Figures 3a and b). On the contrary, reduced levels of phosphorylated p38, as well as PGC-1*α*, TFAM and COX IV were observed under similar conditions in H1299-DN and DKO-MEF cells (Figures 3a and b). Moreover, we noticed the increase in mRNA levels of PGC-1*α* and COX5b in H1299-EV and WT-MEF cells grown under glucose-limiting conditions or upon activation of AMPK as compared with their respective counterparts (Figure 3c). Under glucose-limiting conditions, activities of respiratory complex I and citrate synthase were increased, which were further elevated by AICAR in H1299-EV and WT-MEFs (Figures 3d and e). However, irrespective of glucose concentration and AICAR treatment, activity of these enzymes remained unaltered in H1299-DN and AMPK-DKO cells (Figures 3d and e). Relative ATP level was increased upon AICAR treatment in H1299-EV and WT-MEFs under glucose-limiting conditions, which was unaffected in H1299-DN and AMPK-DKO cells (Figure 3f).

To confirm whether p38 is involved in the regulation of PGC-1*α*, we used p38-specific inhibitors SB203580 and SB220025. We observed that both these inhibitors diminished phosphorylation of p38 though the level of p-AMPK was unaffected in H1299 cells. Reduced levels of PGC-1*α*, TFAM, COX IV and Cyt C were detected in H1299 cells treated with these inhibitors even upon activation of AMPK (Figure 3g). Moreover, we noted that inhibition of p38 caused cell death under metabolic stress condition as evident by reduced ATP level in H1299 cells treated with AICAR and p38 inhibitors (Supplementary Figures S6a–d), which is likely because of disruption in energy homeostasis mediated by AMPK. To verify whether AMPK-induced activation of p38 is mediated by mTOR, we checked phosphorylated p38 level in H1299 cells grown under glucose-limiting conditions in the presence or absence of p38 inhibitor SB203580. We observed that rapamycin treatment did not influence the level of phosphorylated p38 (Supplementary Figure S7), though the levels of molecules involved in mitochondrial biogenesis were increased (Supplementary Figure S7), suggesting that AMPK-mediated activation of p38 is independent of mTOR. Collectively, these results suggest that activation of p38 is required for the AMPK-dependent mitochondrial biogenesis.

### AMPK controls metabolic adaptation under glucose limitation

**Figure 4:**
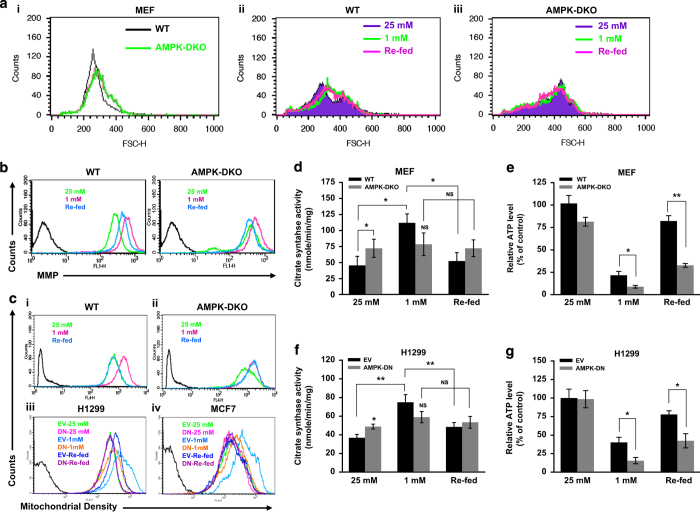
AMPK controls metabolic homeostasis in cancer cells. (**a**) WT and DKO MEFs were cultured in DMEM containing either 25 or 1 mM glucose and 2 mM glutamine for 24 h. The medium was replaced with fresh medium containing 25 mM glucose and further grown for 24 h. Size of these cells were determined by flow cytometry. Relative size of WT and AMPK-DKO cells under normal metabolic condition (25 mM glucose) (i), change in cell size of WT-MEF (ii) and DKO-MEF (iii), cultured in the presence of 25 mM glucose, 1 mM glucose or re-fed with 25 mM glucose. (**b**) Mitochondrial membrane potential (MMP) in MEF cells grown under the conditions mentioned in **a**. DiOC6 was used to determine MMP by flow cytometry. (**c**) MEF and cancer cells (H1299-DN and MCF7-DN) were grown under the conditions mentioned above. Mitochondrial density was determined by flow cytometry using probe MitoTracker Red FM. Mitochondrial density in WT (i), AMPK-DKO MEFs (ii), H1299 (EV and DN) (iii) and MCF7 (EV and DN) cells (iv). (**d**–**f**) MEFs (WT and DKO), H1299 (EV and DN) cells were cultured under the indicated conditions for 24 h. Citrate synthase activity (**d** and **f**) and relative ATP levels (**e** and **g**) were determined in these cells as described in Materials and Methods. Values are represented as mean±S.D. *P<0.05 and **P<0.01 denote significant differences between the groups.

To explore whether AMPK is involved in maintaining metabolic and energy homeostasis, we cultured MEF cells in 25 or 1 mM glucose for 24 h, and these were replenished with 25 mM glucose thereafter. We noticed that AMPK-DKO cells are larger in size as compared with WT cells cultured in 25 mM glucose (Figure 4a-i). An increase in the cell size of WT-MEF was observed upon switching them to 1 mM glucose, while replenishing glucose-restored cell size (Figure 4a-ii). On the other hand, cell size did not alter in DKO cells under identical conditions (Figure 4a-iii). This observation suggests the involvement of AMPK in regulating cell size under metabolic stress.

Subsequently, a prominent increase in mitochondrial membrane potential (MMP) and density was observed in WT cells under glucose-limiting conditions (Figures 4b (left) and c-i), which was restored upon glucose replenishment (Figures 4b (left) and c-i). AMPK-DKO cells exhibited an increase in MMP and mitochondrial density even under glucose-abundant conditions while a moderate increase in mitochondrial density was observed under glucose-limiting conditions (Figures 4b (right) and c-ii). However, upon replenishing glucose to DKO cells cultured in glucose-limiting conditions, MMP and mitochondrial density were not restored (Figures 4b (right) and c-ii). Similar findings were also observed in H1299 and MCF7 cells stably transfected with either empty vector or DN-AMPK (Figures 4c-iii and c-iv). Moreover, enhanced mitochondrial citrate synthase activity was detected in WT-MEFs and H1299-EV cells under glucose-limiting conditions, which decreased upon replenishing glucose (Figures 4d and f). However, no significant change was observed in citrate synthase activity in AMPK-DKO and H1299-DN cells (Figures 4d and f). Further, we checked whether cells are able to maintain ATP level under the condition of fluctuating glucose concentrations. We found that relative ATP levels were decreased in WT-MEF and H1299-EV cells under glucose-limiting conditions, which was increased upon replenishing glucose (Figures 4e and g). In AMPK-DKO and H1299-DN cells, glucose-limiting condition caused a reduction in the ATP level. Upon glucose replenishment, however, there was relatively slight increase in ATP level (Figures 4e and g).

**Figure 5:**
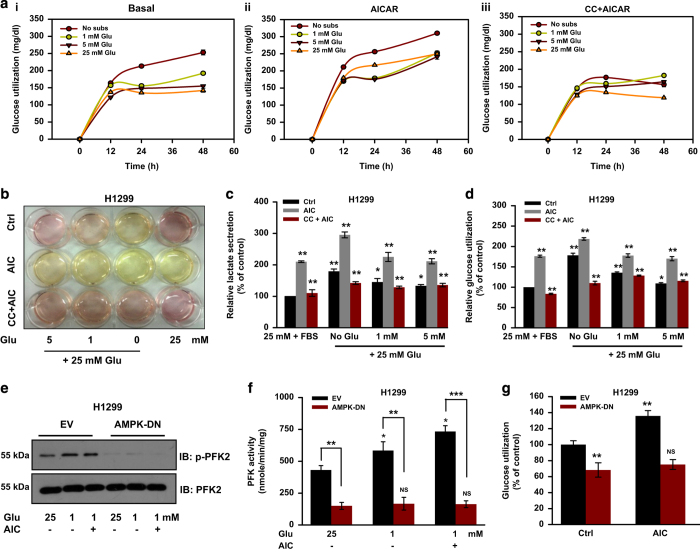
AMPK promotes glycolysis by activating PFK2. (**a**) H1299 cells were grown in DMEM without glucose and glutamine, and supplemented with 1 mM, 5 mM and 25 mM glucose. Cells were simultaneously treated with 0.5 mM AICAR and/or 10 *μ*M compound C for 24 h. Fresh medium (DMEM containing 25 mM glucose and 10% FBS) was added after 24 h and further grown for indicated time points. Time-dependent glucose utilization in H1299 cells under basal (i), AICAR treated (ii) or in the presence of compound C plus AICAR (iii). (**b**) Representative photographs of culture medium from H1299 cells grown under above-mentioned conditions for 24 h. (**c**, **d**) lactate secretion (**c**) and glucose utilization (**d**) in H1299 cells grown under the conditions used in **a**. (**e**) Immunoblots showing phosphorylated and basal levels PFK2 in H1299 (EV and DN) cells grown under indicated conditions for 24 h. (**f**) PFK activity in H1299 (EV and DN) cells grown under indicated conditions for 24 h. (**g**) Relative glucose utilization in H1299-EV and H1299-DN cells treated with or without AICAR for 36 h. Values are represented as mean±S.D. *P<0.05, **P<0.01 and ***P<0.001 denote significant differences between the groups.

AMPK is known to induce glycolysis by directly phosphorylating 6-phosphofructo-2-kinase (PFK2) in muscles, which by activating PFK, enhances glycolytic rate.^28,29^ To explore role of AMPK in metabolic homeostasis, we cultured H1299 cells in the presence of varying concentrations of glucose with or without AICAR and compound C, and after 24 h, medium was replenished with fresh medium containing 25 mM glucose. We noticed that cells grown in no or less glucose exhibited increased glucose utilization in a time-dependent manner upon replenishing glucose (Figure 5a-i). AICAR treatment further enhanced glucose utilization which was reduced by compound C under these conditions (Figures 5a-ii and a-iii). Lactate secretion (Figures 5b and c) was proportionally associated with utilization of glucose under these conditions (Figure 5d). Increased phosphorylation of PFK2 with concomitant increased activity of rate-limiting enzyme PFK was detected in H1299-EV cells grown in glucose-limiting conditions and/or treated with AICAR (Figures 5e and f). However, no change in the phosphorylated level of PFK2 and PFK activity was observed in H1299-DN cells under identical conditions (Figures 5e and f). A significant decrease in the glucose utilization was observed in H1299-DN cells treated or untreated with AICAR as compared with H1299-EV cells (Figure 5g). Our results suggest that AMPK mediates metabolic and energy homeostasis in cancer cell under glucose-limiting conditions by promoting both glycolytic as well as mitochondrial metabolism.

## Discussion

AMPK, under nutritional stress, maintains energy balance by minimizing energy expense and accelerating ATP generation to restore its level in favor of cell survival.^30^ Role of AMPK in cancer is not very clear as it has been reported to exhibit both tumor-suppressing as well as tumor-promoting function.^31–33^ Considering the heterogeneity and physiological stress in tumor microenvironment, it is inevitable for cancer cells to evolve a mechanism to overcome/adapt to various stresses to maintain survival. Our study highlights the important role of AMPK in maintaining energy and metabolic homeostasis, and in promoting cell survival under glucose-limiting conditions.

Role of AMPK in cell survival and metabolic homeostasis in normal (noncancerous) tissues has already been well understood.^11^ However, its role in cancer remains paradoxical. Jeon *et al*.^33^ have shown that activation of AMPK is required for the survival of cancer cells by maintaining redox balance and by promoting fatty acid oxidation. It has also been shown that mTOR inhibition prolongs cell survival under glucose-deprived conditions in TSC2 null cells.^19^ Consistent with these reports, our results demonstrate that AMPK-mediated cell survival requires inhibition of mTOR, as rapamycin promotes survival under both AMPK-activated or -inactivated conditions. Under glucose-limiting conditions, activation of mTOR would perturb energy homeostasis as mTOR mainly regulates anabolic processes.^19^ Thus, inhibition of mTOR is essential to maintain energy and metabolic homeostasis under metabolic stress. Our data show that mTOR inhibition augments ATP levels which positively correlate with cell survival even in the absence of AMPK. As AMPK and mTOR have antagonistic role in cells, inhibition of mTOR might be required for AMPK-mediated metabolic homeostasis. Importantly, we show that AMPK facilitates metabolism of non-glucose substrates, specially glutamine and lactate, to maintain cell survival. This could be an alternative mechanism to restore cellular ATP level under glucose-limiting conditions. It has been shown that in the absence of glucose, glutamine serves as a major energy source in cancer cells.^19,34^ Similarly, cancer cells can also metabolize lactate and fatty acids, under metabolic stress, to derive ATP.^33,35^ Our results demonstrate that activation of AMPK is required for the metabolism of these substrates as cells lacking functional AMPK fail to grow in the presence of these substrates. This alternative metabolism can provide survival advantage to cancer cells under glucose-limiting conditions.

To generate more ATP, enhanced mitochondrial function is required. It has been reported that, under chronic energy deprivation, mitochondrial activity is enhanced in muscle cells.^17,22^ CPT1C, a mitochondrial enzyme, has been shown to maintain survival of breast cancer cells treated with metformin under metabolic stress.^36^ Interestingly, mitochondrial biogenesis and its function are elevated in response to chronic energy deprivation in muscle.^17^ In agreement with these findings, we report that mitochondrial activity is enhanced in cancer cells under glucose-limiting conditions. Recent correlative studies suggest that activation of AMPK is associated with increase in levels and activity of mitochondrial enzymes^16^ and also in mitochondrial biogenesis,^8^ in rat skeletal muscles. Concurrent with these reports, we observed that enhanced mitochondrial biogenesis and activation of AMPK is required for maintaining mitochondrial OXPHOS capacity to sustain ATP levels in cancer cells under metabolic stress. Our results indicate that activation of AMPK enhances expression of PGC-1*α* and its target genes in cancer cells under metabolic stress. PGC-1*α* is a major transcription coactivator protein required for mitochondrial biogenesis. PGC-1*α* promotes oxidative metabolism, mitochondrial biogenesis and facilitates glutamine metabolism to support proliferation^37–39^ and metastasis,^4^ although very little is known about the regulation of PGC-1*α* in cancer cells. Our data suggest that expression of PGC-1*α* can be regulated by AMPK. Previously, it has been reported that rapamycin increases expression of PGC-1*α*.^40^ We also observed that rapamycin treatment increases expression of the PGC-1*α* independent of AMPK suggesting that mTOR inversely correlates with PGC-1*α* expression and mitochondrial biogenesis. It is likely that AMPK could regulate mitochondrial biogenesis via inhibition of mTOR. In addition, we demonstrate that activation of AMPK with concomitant inhibition of mTOR is a prerequisite for maintaining cellular ATP level as well as mitochondrial function.

Stress kinase p38MAPK has been shown to regulate mitochondrial biogenesis by regulating expression of PGC-1*α* in muscles^24,25^ and AMPK can activate p38.^41^ The role of both AMPK and p38 in tumorigenesis is a paradox.^42–44^ Activation of both these kinases has been shown to inhibit cell proliferation.^31,45^ On other hand, it has also been reported that p38 signaling cascades augment cell survival and growth.^46,47^ Our findings indicate that p38 activity is required for AMPK-mediated cell survival under metabolic stress; and as these two together can regulate PGC-1*α*, we sought to explore the connection between the two. Our results suggest that p38 is a downstream activator of AMPK-induced PGC-1*α* expression, and inhibition of p38 does not affect AMPK activation but reduces the levels of PGC-1*α* and TFAM. As both of these are stress kinases, we reasoned that increased activity of these kinases is required to maintain cancer cell survival under glucose-limiting conditions.

Adaptation to metabolic changes and nutrient availability in tumor microenvironment is an important biological process for cell survival.^32,33^ Our data show that cells respond to the nutrient availability by regulating mitochondrial activity which increases under limited glucose availability and can be restored to normal levels upon re-feeding cells with glucose. Interestingly, inhibition of AMPK abrogates cells ability to adapt to fluctuation in glucose levels thereby perturbing metabolic/energy homeostasis leading to cell death. Another important aspect of metabolic homeostasis is to speed up the rate of metabolic reaction to regenerate more ATP and increase in glycolysis under metabolic stress is considered as an adaptive response of cells for maintaining energy homeostasis.^28,29^ We also noted that, AMPK by phosphorylating PFK2, enhances glycolysis. AMPK-dependent increase in glycolysis is a homeostatic mechanism to rapidly restore the energy pool of the cells under metabolic stress in heart and muscle.^28,29^

**Figure 6:**
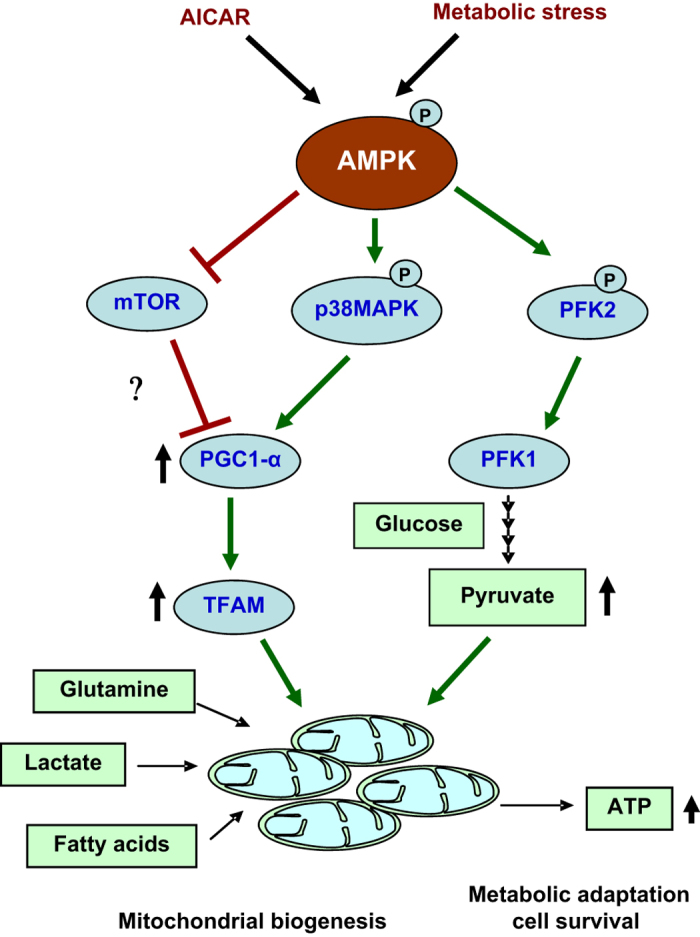
Schematic representation of the mechanism of AMPK-mediated cell survival. AMPK is activated under glucose-limiting conditions as well as by AICAR. AMPK, by increasing p-p38 and by inhibiting mTOR, regulates expression of PGC-1*α* which controls mitochondrial biogenesis in cancer cells and allows oxidative metabolism of non-glucose carbon sources (glutamine, lactate and fatty acids) to generate ATP. To restore ATP pool, AMPK enhances metabolic rate by activating PFK2.

Conclusively, above-stated results indicate that AMPK is an important proximal signaling step for regulating mitochondrial biogenesis and cell survival under metabolic stress. We, for the first time, show that AMPK can regulate mitochondrial biogenesis in cancer cells to promote metabolism of non-glucose carbon sources via regulating p38/PGC-1*α* (Figure 6). Together, our study provides mechanistic insight into the protective role of AMPK in cancer cells under glucose-limiting conditions.

## Materials and Methods

### Cell lines and culture conditions

H1299 and MCF7 cells were obtained from ATCC (Manassas, VA, USA) and maintained in our in-house repository. H1299 cells expressing dominant-negative (AMPK-DN) form of AMPK-*α*1 subunit were generated by transfecting pCDNA3-AMPK*α*1-DN or empty vector (pCDNA3) only. AMPK-*α*1/*α*2^+/+^ (WT) and AMPK-*α*1/*α*2^−/−^ (DKO) SV40-immortalized MEFs have been described previously.^32^ All the cells were grown in DMEM containing 2 mM/l glutamine supplemented with 10% FBS and 1% penicillin/streptomycin (Life Technologies, Carlsbad, CA, USA). For experimental purpose, culture conditions were defined as, (i) glucose-abundant condition: 25 mM glucose and 10% FBS; (ii) glucose-limiting condition: 1 mM glucose, 2 mM glutamine and 10% FBS; (iii) glucose-deprived condition: 2 mM glutamine and 1% FBS.

### Chemicals and reagents

AICAR, compound C, glucose, glutamine, palmitic acid (sodium salt), lactate, oligomycin, rapamycin, 6-diazo-5-oxo-L-norleucine (DON) and epigallocatechin 3-gallate (EGCG) were purchased from Sigma (St. Louis, MO, USA). SB203580 and SB220025 were obtained from Calbiochem (San Diego, CA, USA). Antibodies for p-AMPK-Thr 172 (Cat# 2535, Dil 1:1000), p-p70S6K-Thr 389 (Cat# 9234, Dil 1:1000) and p-p38-Thr 180/Tyr 182 (Cat# 9211, Dil 1:1000) were purchased from Cell Signaling Technology (Danvers, MA, USA). Antibodies for pACC-Ser 79 (Cat# SC271965, Dil 1:1000), ACC (Cat# SC30212, Dil 1:1000), p70-S6Kinase (Cat# SC230, Dil 1:1000), AMPK (Cat# SC25792, Dil 1:1000), PGC-1*α* (Cat# SC13067, Dil 1:1000), TFAM (Cat# SC376672, Dil 1:2000), p-p38-Tyr 172 (Cat# SC7973, Dil 1:1000), p38 (Cat# SC535, Dil 1:1000), Survivin (Cat# SC17779, Dil 1:1000), Bcl-2 (Cat# SC7382, Dil 1:1000), Bcl-XL (Cat# SC7195, Dil 1:1000), COX IV (Cat# SC292052, Dil 1:1000), Cyto C (Cat# 7159, Dil 1:1000), pPFK2-Ser 466 (Cat# SC32966, Dil 1:1000), PFK2 (Cat# SC10090, Dil 1:1000), c-Myc (9E10) (Cat# SC40, Dil 1:1000), HSP60 (Cat# SC13115, Dil 1:2000), GAPDH (Cat# SC20357, Dil 1:1000) were from Santa Cruz Biotechnology (Dallas, TX, USA).

### Determination of cell viability and cell size

Cell viability was assessed as described earlier by propidium iodide (PI) staining using flow cytometry.^19^ Briefly, cells were washed, trypsinized and pellet was collected by centrifuging at 1000 ×*g* for 5 min. After washing with sterile PBS, the cells were incubated with PI (200 ng/ml in PBS) for 10 min. PI fluorescence was acquired by flow cytometer using FL2 filter (FACS Calibur, BD Bioscience, San Jose, CA, USA). Cells positive with PI were counted as dead cells. Data were analyzed using Cell Quest Pro software (Becton Dickinson, San Jose, CA, USA) for 2.5×10^4^ cells. The experiments were performed in triplicate and repeated at least once. Alternatively, cell number was counted using hemocytometer for accessing cell proliferation. Change in cell size was determined by measuring forward scatter (FSC) via flow cytometry as described previously.^19^

### Long-term cell survival assay

Cells were seeded at a density of 1×10^3^/well in 12-well culture plates, and were allowed to adhere for 24 h at 37 °C. Cells were treated as per the experimental requirement. Medium was replaced with fresh medium and further grown for 1 week. Crystal violet staining was performed as described.^48^

### siRNA transfection

siRNA against AMPK-*α*1/2 and AMPK-*α*1 were purchased from Santa Cruz Biotechnology. Transfection was done using Lipofectamine 2000 (Life Technologies) according to the manufacturer’s instructions. Transfection efficiency was assessed by simultaneously transfecting pEGFPN1 plasmid. Immunoblotting was performed to ensure inhibition of respective gene expression.

### Cell lysate preparation and immunoblotting

Cell lysates were prepared and subjected to immunoblotting as described previously.^48^ Whenever required, membranes were washed thoroughly with TBS (Tris-buffered saline) and re-probed with desired antibodies.

### Quantitative real-time PCR analysis

RNA from cells was extracted using TRIzol reagent (Invitrogen, Carlsbad, CA, USA) according to the manufacturer’s instructions. cDNA was prepared using moloney murine leukemia virus reverse transcriptase (MMLV-RT) under the recommended conditions. qRT-PCR was performed on cDNA samples using the Quantitative SYBR green PCR kit (Bio-Rad, Hercules, CA, USA) and was run on the Mastercycler ep realplex Real-time PCR System (Eppendorf, Hamburg, Germany) as described earlier.^4^ Primer sequences used are listed in Supplementary Table S1.

### ATP measurement

ATP level was measured in cells using commercially available ATP bioluminescence kit (Roche, Mannheim, Germany). Briefly, cells were grown in the presence of indicated drugs with or without lactate (10 mM), glucose (1, 5 and 25 mM), glutamine (5 mM), galactose (5 mM) and palmitate (100 *μ*M). Cells were collected and lysed in 100 mM Tris-EDTA (TE) buffer containing 0.01% NP-40 and boiled for 1 min followed by three quick freeze–thaw cycles. Luminescence was recorded for blank as well as for samples. The experiments were done in triplicate and repeated at least twice, and the final values were normalized with total protein contents.

### Mitochondrial density and membrane potential measurement

For measuring membrane potential and mitochondrial density, the cells were resuspended in PBS containing either 100 nM DiOC6 or 50 nM MitoTracker Red FM (Life Technologies), respectively, and incubated for 30 min at 37 °C. The fluorescence of DiOC6 and MitoTracker Red FM were acquired through a 585 nm (FL1) or 650 (FL4) filter in a flow cytometer. Data were analyzed using Cell Quest Pro software (Becton Dickinson) for 1×10^4^ cells.

### Glucose utilization and lactate estimation assay

Cells (3×10^5^) were cultured in DMEM containing 1 or 25 mM glucose. After 24 h, medium was replaced with respective medium containing AICAR or compound C for 24 h, and residual glucose present in the spent medium was monitored using GOD-POD-based glucose assay kit (Spinreact, Girona, Spain). Consumed glucose was estimated by subtracting the remaining glucose in the medium from the initial concentration in control medium (450 mg/dl). All the experiments were performed in triplicate and the values were normalized to total number of cells. Lactate was estimated by using commercially available lactate estimation kit (Spinreact) according to the manufacturer’s protocol. Briefly, cells (3×10^5^) were plated in 12-well tissue culture plates. After 24 h, medium was replaced with DMEM containing 0.2% BSA with 0, 1, 5 and 25 mM glucose either containing 0.5 mM AICAR or 10 *μ*M compound C alone or together for 24 h. The cells were washed with fresh medium and replaced with DMEM containing 25 mM glucose in each well with or without AICAR and compound C for further 48 h. The medium was collected and stored in −20 °C for assay purpose.

### Enzyme assays

Enzyme activity was measured in cells as described earlier with slight modifications.^49^ Briefly, cells cultured under desired experimental conditions were homogenized in hypotonic (20 mM) potassium phosphate buffer (pH 7.5) containing protease inhibitor cocktail (Roche), vortexed and lysed by three cycles of freeze and thaw procedure. Activity of complex I and citrate synthase was determined as described. PFK activity was determined by enzyme-coupled reaction method. Briefly, the enzyme reaction was performed in Tris-Cl buffer (50 mM, pH 8.0) containing ATP (0.1 mM), MgCl_2_ (3.3 mM), NADH (0.1 mM), glyceraldehydes-3-phosphate dehydrogenase (1 U/ml), aldolase (1 U/ml), triose phosphate isomerase (6 U/ml). The reaction was started by adding 3.3 mM fructose 6-phosphate and absorbance was recorded at 340 nm at every 30 s for 10 min. The values were normalized with protein contents.

### Statistical analysis

Unpaired two-tailed Student’s *t*-tests were performed unless otherwise mentioned, using SPSS Software V.12 (San Jose, CA, USA). Data were represented as mean±S.D. unless otherwise specified. Experiments were done in triplicate and most of the experiments were repeated at least once. *P<0.05, **P<0.01, ***P<0.001 denote significant differences between the groups.

## Abbreviations

AIC: AICAR
AMPK: AMP-activated protein kinase
DN: dominant negative
DKO: double knockout
DON: 6-diazo-5-oxo-L-norleucine
EV: empty vector
EGCG: epigallocatechin 3-gallate
Glu: glucose
Gln: glutamine
Lact: lactate
MEF: mouse embryonic fibroblasts
MFI: mean fluorescence intensity
MMP: mitochondrial membrane potential
mTOR: mammalian target of rapamycin
Oli: oligomycin
OXPHOS: oxidative phosphorylation
Palm: palmitate
PGC-1*α*: peroxisome proliferator-activated receptor coactivator-1
Rap: rapamycin
S.D.: standard deviation
WT: wild type.
