# Supplementary material for: AMPK maintains energy homeostasis and survival in cancer cells via regulating p38/PGC-1α-mediated mitochondrial biogenesis
Source: Cell Death Discov. 2015 Dec 21;1:15063–. doi: 10.1038/cddiscovery.2015.63 (PMC4979508; doi:10.1038/cddiscovery.2015.63)
Supplement: Supplementary Information [file cddiscovery201563-s1.pdf]

SUPPLEMENTARY INFORMATION

AMPK maintains energy homeostasis and survival in cancer cells via regulating p38/PGC-1α-mediated mitochondrial biogenesis

Balkrishna Chaube[1], Parmanand Malvi[1], Shivendra Vikram Singh[1], Naoshad Mohammad[1], Benoit Viollet[2], and Manoj Kumar Bhat[1]

National Centre for Cell Science, Savitribai Phule Pune University Campus, Ganeshkhind, Pune, Maharashtra, India 411 007

Supplementary figure S1

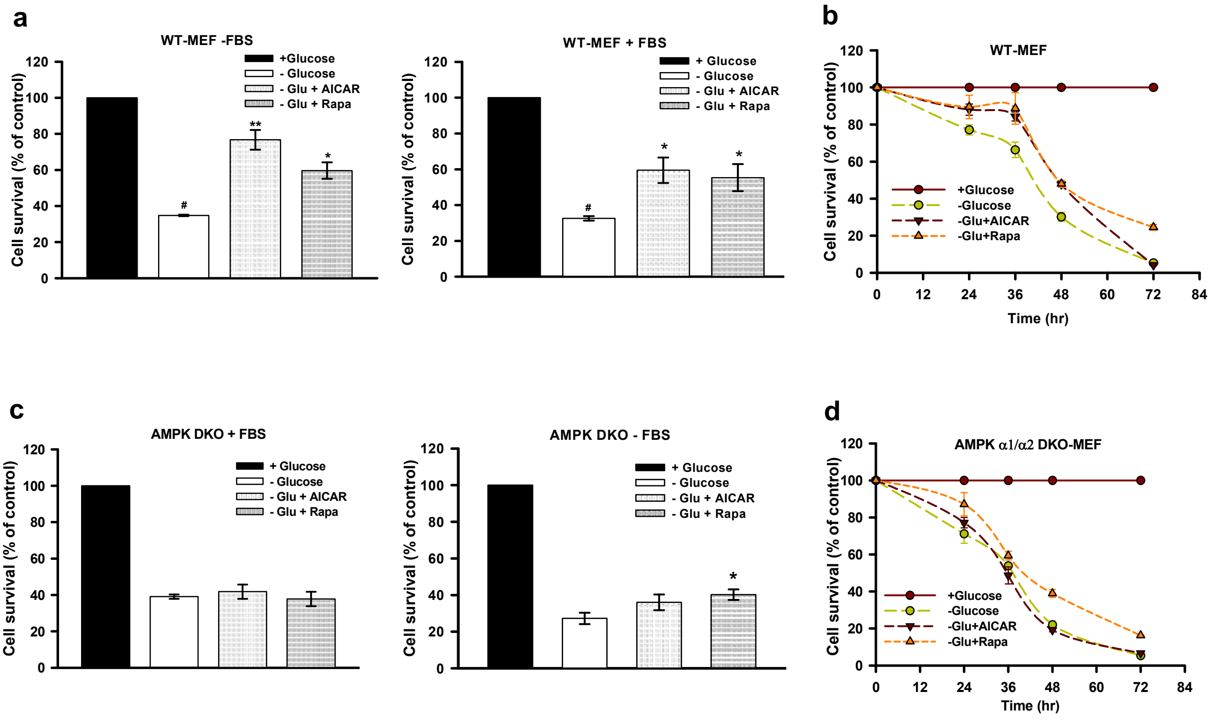

Supplementary figure S1: AMPK protects cell from glucose and FBS deprivation induced cell death. (a) WT-MEFs were cultured in 96 well plates. Cells were grown in DMEM with or without glucose and FBS in presence of absence of 0.5 mM AICAR or 20 nM rapamycin for 72h. Growth was assayed by using MTT assay at indicated time point. **(b)** Time dependent growth curve of WT-MEFs cells cultured under the condition mentioned in (a). **(c)** Cell survival of AMPK α1/α2 double knock out (DKO) cells cultured under condition similar to WT cells mentioned in (a). **(d)** Time dependent growth curve of WT-MEFs cells cultured under the condition mentioned in (a). All values are represented as mean ± SD.

Supplementary figure S2

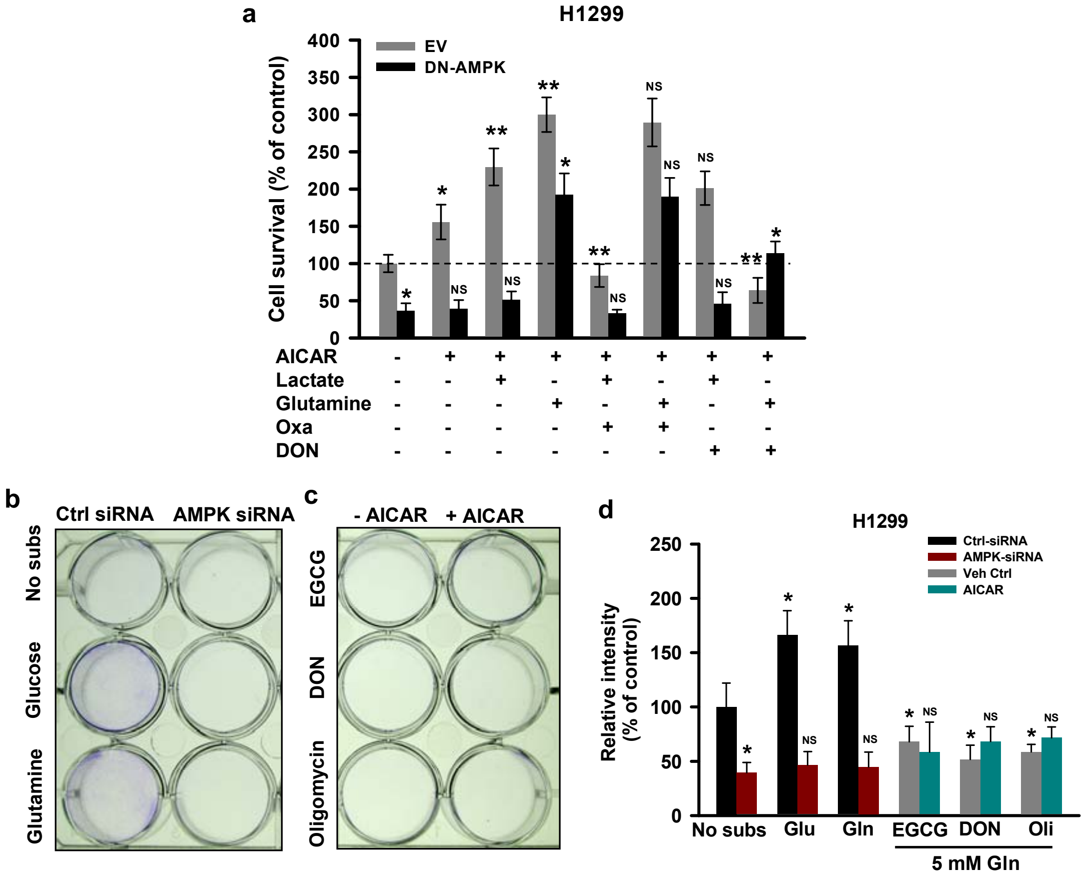

Supplementary figure S2: Inhibiting lactate and glutamine metabolism in cancer cells abrogate AMPK mediated cell survival. (a) Cell survival in H1299-EV and H1299-DN cells cultured in DMEM without glucose or in presence of absence of 10 mM lactate and 5 mM glutamine. Cells were treated with 25 mM oxamate, an inhibitor of enzyme LDH, or 1 mM 6-Diazo-5-oxo-L-norleucine (DON) for 48 h either alone or in presence of AICAR. **(b)** Long term survival of H1299 cells transfected with either control siRNA or AMPKα1/α2 siRNA and cultured in DMEM containing either glucose or glutamine. Cells were grown for 48 h and medium was replaced with fresh medium containing 25 mM glucose and 2 mM glutamine and further grown for 1 weeks. (**c** and **d**) H1299 cells were grown in glucose-free DMEM containing 5 mM glutamine. Cells were treated with 50 µM epigallocatechin 3-gallate (ECEG), 1 mM 6-diazo-5-oxo-L-norleucine **(**DON) or 1 µM oligomycin in presence or absence of AICAR for 48 h. medium was replaced with drug free medium and further grown for 1 week. Representative image of plates stained with crystal violet (c) and quantitative representation of relative intensity (d).

Supplementary figure S3

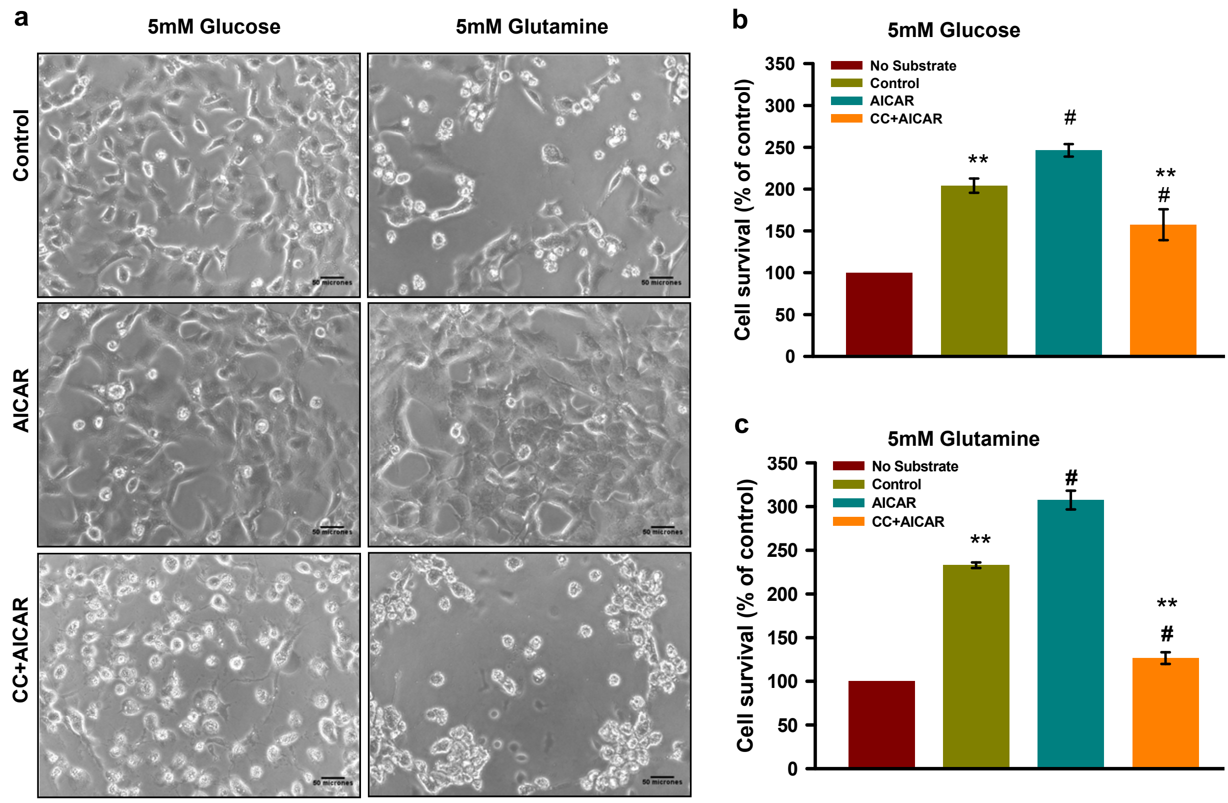

Supplementary figure S3: **AMPK regulates glucose metabolism under metabolic stress (a-c)** H1299 cells were cultured in DMEM containing either 5 mM glucose or 5 mM glutamine with or without AICAR and compound C for 48 h. (**a**) Representative image of cells, (**b** and **c**) represents percent cell survival after the treatment of indicated reagents for 48 h. (Scale bar = 50 μM). All values are represented as mean ± SD.

Supplementary figure S4

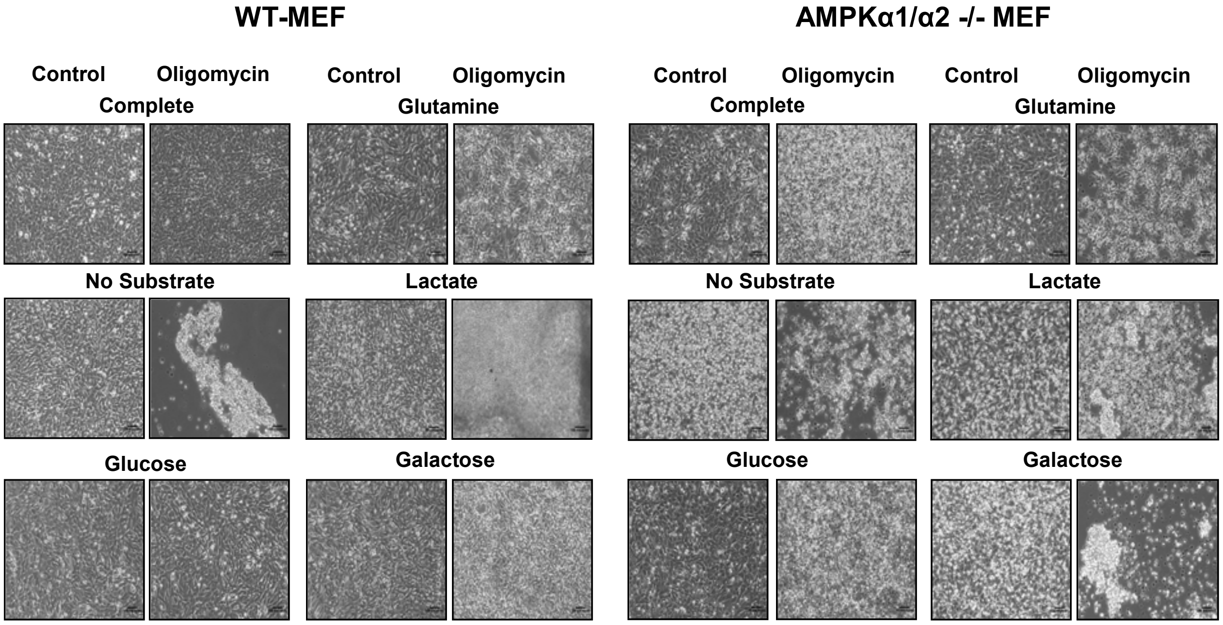

Supplementary figure S4: **AMPK regulated mitochondrial metabolism of non-glucose carbon source like lactate and glutamine.** Representative image showing the morphology and cell survival of WT and AMPK-DKO cells cultured in presence or absence of 5 mM glucose, 5 mM glutamine, 5 mM galactose and 10 mM lactate with or without 10 µM oligomycin for 48 h. (Scale bar = 50 μM)

Supplementary figure S5

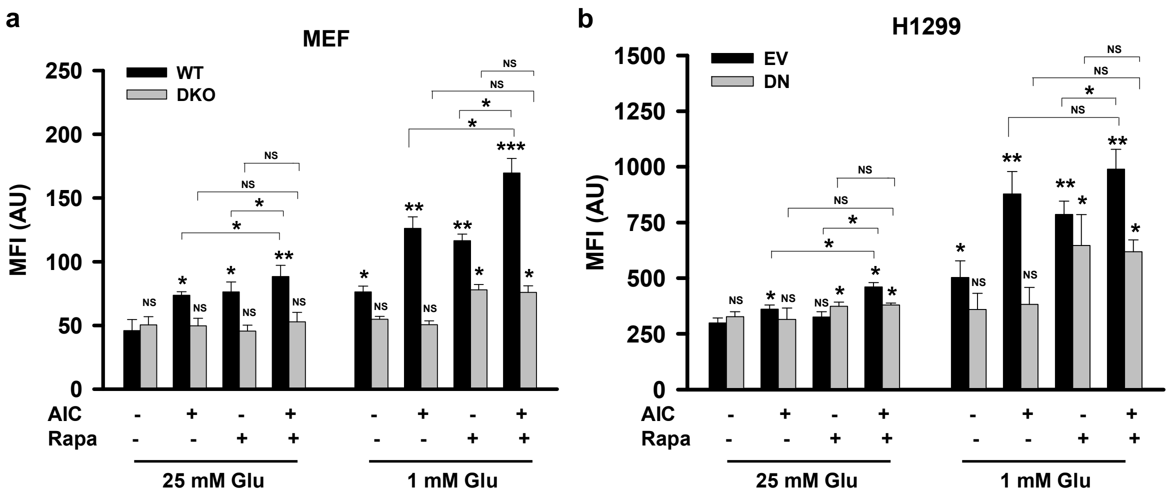

Supplementary figure S5: **AMPK increases mitochondrial density in cancer cells by suppressing mTOR.** (**a**) MEF (WT and AMPK-DKO) and (**b**) H1299 (EV and DN) cells were cultured in either 25 mM or 1 mM glucose, and 2 mM glutamine in presence or absence of 0.5 mM AICAR or 20 nM rapamycin either alone or together for 24 h. Mitochondrial density was measured using Mitotracker Red FM by flow cytometry. All values are represented as mean ± SD.

Supplementary figure S6

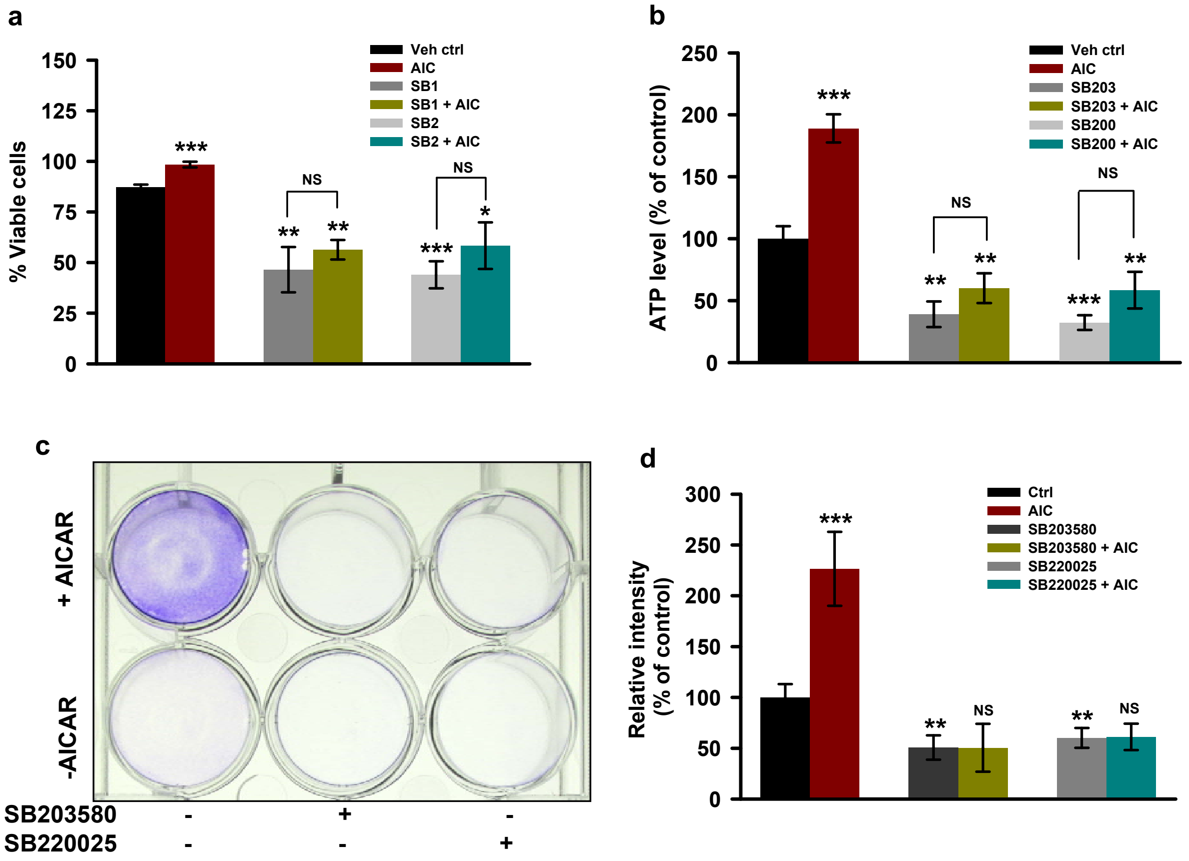

Supplementary figure S6: **Inhibition of p38MAPK prevents AMPK induced cell survival under metabolic stress. (a)** H1299 cells were cultured in DMEM containing 1 mM glucose and 2 mM glutamine in presence or absence of 25 µM SB203580 and SB220025 either alone or with 0.5 mM AICAR for 48 h. Cell viability was accessed by PI staining via flow cytometry. (**b**) ATP level in H1299 cells grown under the condition mentioned in (a). (**c** and **d**) Long term cell survival in H1299 cells grown in presence of 25 µM SB203580 and SB220025 either alone or with 0.5 mM AICAR for 24 h. Medium was replaced with drug free medium and further grown for 1 week. Representative image showing long term survival of H1299 cells (c), quantitative representation of intensity of stained cells (d). All values are represented as mean ± SD.

Supplementary figure S7

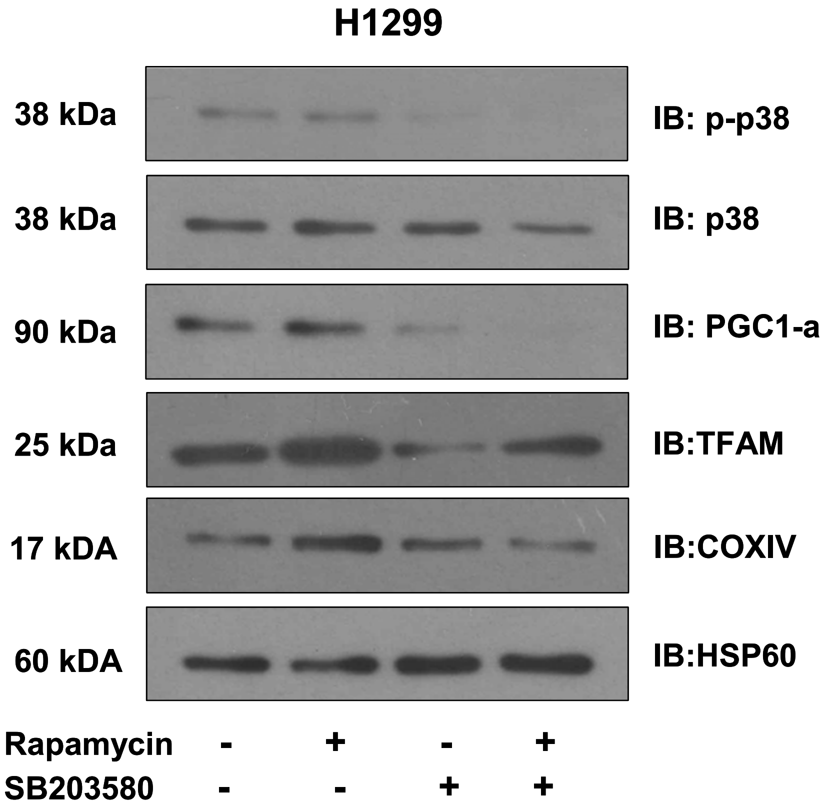

Supplementary figure S7: R**apamycin increases levels of molecules involved in mitochondrial biogenesis.** Immunoblots showing the levels of indicated molecules in H1299 cells cultured in glucose limiting condition with or without 20 nM rapamycin and 10 μM p38 specific inhibitor SB203508 for 24 h.

Supplementary Table S1

Gene	Sequence
**h Actin**	**F 5'-CATGTACGTTGCTATCCAGGC-3'**
	**R 5'-CTCCTTAATGTCACGCACGAT-3'**
**hPGC1α**	**F 5'-CTGCTAGCAAGTTTGCCTCA-3'**
	**R 5'-AGTGGTGCAGTGACCAATCA-3'**
**hCOX5b**	**F 5'-ATGGCTTCAAGGTTACTTCGC-3'**
	**R 5'-CCCTTTGGGGCCAGTACATT-3'**
